# Determination of the Antifungal, Antibacterial Activity and Volatile Compound Composition of *Citrus bergamia* Peel Essential Oil

**DOI:** 10.3390/foods12010203

**Published:** 2023-01-03

**Authors:** Nur Cebi, Azime Erarslan

**Affiliations:** 1 Food Engineering Department, Chemical-Metallurgical Faculty, Yıldız Technical University, Istanbul 34210, Turkey; 2 Bioengineering Department, Chemical-Metallurgical Faculty, Yıldız Technical University, Istanbul 34210, Turkey

**Keywords:** GC–MS, volatile composition, *C. bergamia*, antimicrobial, essential oil, post-harvest fungi

## Abstract

Safe and health-beneficial citrus oils can be employed as natural preservatives, flavorings, antioxidants, and as antibacterial and antifungal agents in a wide variety of food products. In this research, using GC–MS methodology, the major volatile composition of *Citrus bergamia* EO, obtained by hydro-distillation, was determined to consist of limonen (17.06%), linalool (46.34%) and linalyl acetate (17.69%). The molecular fingerprint was obtained using FTIR spectroscopy. The antibacterial effect of *C. bergamia* EO at different concentrations (0.5, 1, 2.5 and 5 µg/mL) was tested against different pathogen species (*Salmonella typhimurium, Bacillus cereus, Staphylococcus aureus, Escherichia coli, Listeria monocytogenes*), based on disc diffusion assay. The in vitro antifungal activity of *C. bergamia* EO oil against *Aspergillus niger* and *Penicillium expansum* was evaluated using agar disc diffusion assay. Clear inhibition zones were formed by *C. bergamia* EO against selected species of pathogens. Almost all of the concentrations were revealed to have antifungal activity against selected fungal pathogens. The highest inhibition rate of *A. niger* at 6 incubation days was 67.25 ± 0.35 mm with a 20 µL dose, while the growth in the control was 90.00 ± 0.00 mm. In addition, the highest inhibition rate of *P. expansum* was 26.16 ± 0.76 mm with a 20 µL dose, while the growth was 45.50 ± 2.12 mm in the control fungus. A higher antifungal effect of *C. bergamia* EO against *P. expansum* was obtained. It was observed that the growth of fungi was weakened with increasing concentrations (5, 10, 15 and 20 µL dose) of *C. bergamia* EO. Statistically significant (*p* < 0.05) results were obtained for the antibacterial and antifungal effects of *C. bergamia* EO. The findings from the research may shed light on the further use of *C. bergamia* EO obtained from peels in innovative food engineering applications in order to maintain food quality, food safety, and food sustainability.

## 1. Introduction

Essential oils obtained from plants are natural valuable products that have economic and commercial importance in industrial applications. With their unique volatile compounds, essential oils have pioneering importance in foods, drinks, perfumeries, pharmaceuticals and cosmetics [1]. Several studies have documented the fact that essential oils can be employed as natural preservatives, flavorings, antioxidants, and antibacterial and antifungal agents in a wide variety of food products. In particular, citrus oils have been found to have tremendous applications in the area of food production because of their safe and health-beneficial properties [2]. 

To date, essential oils have attracted attention in both the academic field and industrial applications, due to their properties, including volatility and safety. The main active EO components are phenols, terpenes, aldehydes and ketones, whose actions are directed against the cytoplasmic membranes of target microorganism cells [3]. Factors found to influence essential oil composition have been explored in several studies, and a number of researchers have demonstrated that genetic variation, geographical location of the plants, seasonal variations, and climate change are some of the major factors that determine the uniqueness and chemistry of essential oils [1]. The chemical composition of essential oils determines their antifungal, antibacterial, antioxidant and insecticidal capabilities. Consequently, various studies have been dedicated to exploring the volatile composition of essential oils [4].

Today, there is a growing interest, in both academic institutions and in industry, in determining the safe and green capabilities of using essential oils to combat and control pests and diseases in agriculture [5]. Economic losses can result from the growth of fungal microorganisms in edible agricultural products, causing plant diseases and food spoilage. In agriculture, fruit and vegetables are often exposed to microbial contamination from pathogenic fungi during post-harvest storage [6,7]. Due to the widespread application of chemical fungicides, most pathogenic strains have developed a resistance to them. In addition, there are reports of effects, such as carcinogenesis, residual toxicity and environmental pollution, caused by the continued use of such fungicides [8]. In order to minimize the undesirable side effects of synthetic fungicide applications, researchers have focused on evaluating alternative natural biofungicides. With a view to ensuring consumer safety when consuming fruit and vegetables protected by using synthetic fungicides, natural extracts (EOs) have been investigated as healthy and non-toxic alternatives for the past few decades [9].

Recently, in many countries around the world, there has been an apparent increase in demand for organic products that have not been treated with agrochemicals, especially after harvest. Therefore, essential oils can be evaluated as safe and reliable antimicrobial and antifungal agents to effectively manage major post-harvest diseases. Related to the demand for natural and reliable control agents, essential oils have become prominent as a means of safe alternatives to synthetic fungicides [10].

*C. bergamia*, also known as “Bergamot”, is a plant belonging to the Rutaceae family, and takes the form of a hybrid of bitter orange and lemon. The main producer countries are Italy, countries in East Africa, Ivory Coast, Argentina, Brazil and Turkey. The essential Bergamot oil (*C. bergamia* EO) has commercial use in the perfumery and essence industries due to its favorable aroma and fragrance properties. Other usage areas can be listed as the pharmaceutical industry and the food industry. As is recognized as safe, *C. bergamia* EO can be used in the latter as a flavoring agent in a wide range of foodstuffs, such as liqueurs, tea, coffee, ice cream, confectionery and drinks [11]. 

The essential oil quality varies depending on the geographical origin, climate change, seasonal factors and soil properties. Consequently, the volatile composition, and the bioactive, antifungal and antibacterial properties exhibit changes based on these factors. The above-mentioned functional properties of essential oils determine the industrial quality and their use in high-value commercial products. Additionally, at the present time, waste management and valorization are important problems. In this context, the determination of the chemical composition of the waste parts of fruits, such as peels, is gaining growing importance. To the best of our knowledge, this study is the first attempt to comprehensively evaluate the antimicrobial, antifungal, molecular and volatile properties of *C. bergamia* peel essential oil, obtained by the hydro-distillation of fresh bergamot peels (Turkey, Hatay). There is a need for thorough studies in which the specific properties of *C. bergamia* peel essential oil is investigated in detail using robust analytical techniques. The aim of this study was to first evaluate the antifungal activity of *C. bergamia* peel essential oil from Turkey by in vitro methods, against *A. niger* and *P. expansum* fungal pathogens and *E. coli, L. monocytogenes, B. cereus* and *S. aureus* pathogenic bacteria. The second aim of the study was to determine the volatile compound composition of *C. bergamia* essential oil from Turkey (Hatay) using the robust GC–MS technique.

## 2. Materials and Methods

### 2.1. Essential Oil and Chemicals

*C. bergamia* organic fruits originating from Hatay (Turkey) were used in this study. Potato dextrose agar (PDA) and other chemicals were procured from Merck (Darmstadt, Germany). *E. coli* ATCC 8739, *L. monocytogenes* ATCC 13,932, *B. cereus* ATCC 11,778. 

The *S.* Typhimurium ATCC 14,028 and *S. aureus* ATCC 6538 and *A. niger* and *P. expansum* were obtained from Yildiz Technical University, Turkey. Samples were diluted with diethyl ether (Merck-Schuchardt, FRG, GC > 98%) prior to the GC–MS analysis.

### 2.2. Isolation and Identification of Pathogenic Fungi

Fungi were previously isolated from infected apples at the correct stage of maturity to isolate *A. niger* and *P. expansum,* and stored at room temperature until spoilage. These fungi were identified using the available literature, which describes their colony and hyphae morphology, conidial structure and characteristic features [6,12]. Pure cultures were maintained on Potato Dextrose Agar (PDA, Merck, Darmstadt, Germany) at 27 °C with 50 mg/L streptomycin (Merck, Darmstadt, Germany) for 7 days. Spores were collected by filling the media surface with sterile distilled water and gently shaking the plate to remove and separate the spores. A conidial suspension was prepared in sterile Ringer’s solution (Merck, Darmstadt, Germany). Spores were counted and the final inoculum concentration was adjusted to a concentration of 1 × 10^5^ spores/mL per pathogen. Suspensions obtained prior to inoculation were shaken using a vortex mixer for 30 s [13].

### 2.3. In Vitro Antifungal Assay

The disk evaporation method was used to determine the antifungal activity of *C. bergamia* EO against *A. niger* and *P. expansum*. Fungal plugs from a 7-day old, actively growing, culture, 6 mm in diameter, were inoculated into petri dishes containing 20 mL of fresh Potato Dextrose Agar (PDA) medium. Diluted essential oils, ranging from 5 to 20 µL/petri, were then adsorbed onto blank antimicrobial disks. The disks were placed on the petri dishes by inverting the petri dishes. Sterile distilled water was used as a control. The petri dishes were covered with parafilm immediately after the addition of the essential oil to allow effective exposure of the vapors to the fungal mycelia, followed by incubation at 27 °C for 7 days. This assay per test pathogen was triplicated to arrive at a statistically sound conclusion [7,9,14].

### 2.4. Linear Polynomial Contrast

The linear relationships between the applied doses of *C. bergamia* essential oil and the growth of the fungal pathogens *(A. niger* and *P. expansum)* were established using a simple linear regression analysis, performed using Origin 6.0 software.

### 2.5. Determination of Antibacterial Activity

The antibacterial activity of *C. bergamia* EO at different concentrations from 0.5 to 5 µg/mL was evaluated by the agar disc diffusion method. Nutrient Agar (NA) medium was used to cultivate the bacteria, and each bacterial suspension was diluted and adjusted to the equivalent of the 0.5 McFarland standard (10^8^ CFU/mL). A sterile 6 mm paper disc, impregnated with 20 µL of each tested *C. bergamia* EO was placed on the surface of the inoculated plates. A disk impregnated with 20 μL of distilled water was used as a negative control. The plates were incubated at 37 °C for 24 h. Microbial inhibition was assessed visually in terms of the diameter of the zones of inhibition surrounding the discs, including the intervertebral discs, and recorded in millimeters according to NCCLS (2015) [15].

### 2.6. Statistical Analysis

The size of the mycelial growth levels (mm) was expressed as the mean of three recordings with their standard deviation. The significance of the mean differences was compared statistically using Student’s *t* test at *p* < 0.05. Experimental data were subjected to one-way analysis of variance (ANOVA) using the JMP (release 6.0.0, SAS) software package.

### 2.7. FTIR Data Acquisition

FTIR measurements were performed by using the ATR accessory of the equipment. Samples were kept in amber vials until analysis at 4 °C. The spectral parameters of resolution and accumulation were selected as 4 cm^−1^ and 16 scans, respectively. Data were acquired and processed by using the OPUS program Version 7.2 (Bruker Gmbh, Bremen-Germany). A quantity of 100 mL of each sample was dripped on the crystal, after which the diamond crystal of the ATR accessory was cleaned with pure ethyl alcohol. An air spectrum was used as a background spectrum prior to each acquisition. 

### 2.8. GC–MS Analysis

#### 2.8.1. *C. bergamia* EO Extraction from Fresh Peels

The essential oil extraction from *C. bergamia* fruits was performed using both a hydro-distillation Clevenger apparatus system and a microwave extraction system (Milestone, Italy). In the traditional hydro-distillation, Clevenger apparatus system was used, *C. bergamia* peel was subjected to hydro-distillation for 2 h with a peel:weight ratio of 1:1. 

The microwave assisted extraction of *C. bergamia* fruit peels was performed in the following procedure. The system included a cooling system outside the microwave oven, with the EO being obtained from a Clevenger-type equipment connected to the oven. The operation temperature was 100 °C, with 600 W power being applied for 35 min. The operation was stopped when no more essential oil was obtained. The water and *C. bergamia* peel weight ratio was selected as 1:1 and 4 mL of essential oil was obtained from 400 g of fresh *C. bergamia* peel. The *C. bergamia* essential oil was stored at 4 °C until the GC–MS analysis.

#### 2.8.2. GC–MS Data Acquisition of *C. bergamia* EO

In the sample preparation, diethyl ether was used as a diluent (1:20). An Rtx-5MS capillary column (30 m × 0.25 mm × 0.25 μm) was used in measurements. The oven temperature increased gradually from 40 °C for 3 min at the beginning of the temperature program, then increased to 100 °C at an 8 °C/min rate, and was then raised to 200 °C at a rate of 5 °C/min, and, finally, to 250 °C at a rate of 10 °C/min. The temperature of the injection block and the flow rate of the carrier gas were 250 °C and 1 mL/ min, respectively. The samples were scanned at a mass range of 35 and 650 (*m*/*z*). The composition of the essential oil was determined by comparing the obtained GC–MS total ion chromatogram with those included in the NIST (National Institute of Standard and Technology) and Wiley Library of GCMS-QP2010 equipment (Shimadzu, Milan, Italy). The quantitative amount of each compound was calculated on the basis of the percentage area of each identified compound chromatogram compared to the areas of total peaks (100%). Samples were scanned three times, and all the peaks detected in at least two of the total ion chromatograms (TIC) were used for the calculation of relative abundance. 

## 3. Results 

### 3.1. Antibacterial Activity Assay

**Figure 1:**
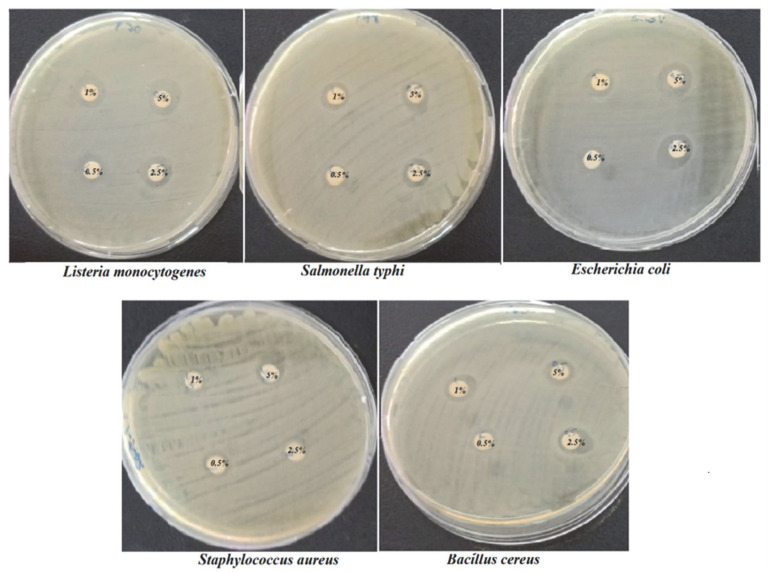
Clear inhibition zones formed by *C. bergamia* EO against different species of pathogens.

In evaluating the antibacterial effect, *C. bergamia* EO was prepared in various concentrations, such as 0.5 µg/mL, 1 µg/mL, 2.5 µg/mL and 5 µg/mL, against *E. coli*, *L. monocytogenes*, *B. cereus*, *S. typhimurium* and *S. aureus* by the disc diffusion method. The results shown in Table 1 indicate that the bacterial strains expressed a varied range of susceptibilities to the actions of *C. bergamia* EOs (Figure 1). 

At a concentration of 0.5 µg/mL, the lowest inhibition rate of 8.50 ± 0.70 mm was observed for *B. cereus*. The lowest inhibition rate for *S. aureus* and the highest inhibition rate for *L. monocytogenes* was achieved at concentrations of 1 and 2.5 µg/mL. At a concentration of 5 µg/mL, the highest inhibition zone of 13.50 ± 0.70 mm was observed for *L. monocytogenes.* As a result of this study, *L. monocytogenes* was identified as the most sensitive bacterium.

### 3.2. In Vitro Antifungal Assay

From Table 2 and Table 3, it is apparent that the *C. bergamia* essential oil showed antifungal properties with regard to both the fungal pathogens. Raising the concentration of essential oil resulted in a higher growth inhibition ratio (%)As a result, almost all of the concentrations were revealed to have antifungal activity against the selected fungal pathogens. Increasing the dose from 5 to 20 µL/petri of *C. bergamia* resulted in a weaker growth of the fungi. The vapors of the essential oil of *C. bergamia* could inhibit the pathogen growth even more at a concentration of 20 µL/petri. 

**Figure 2:**
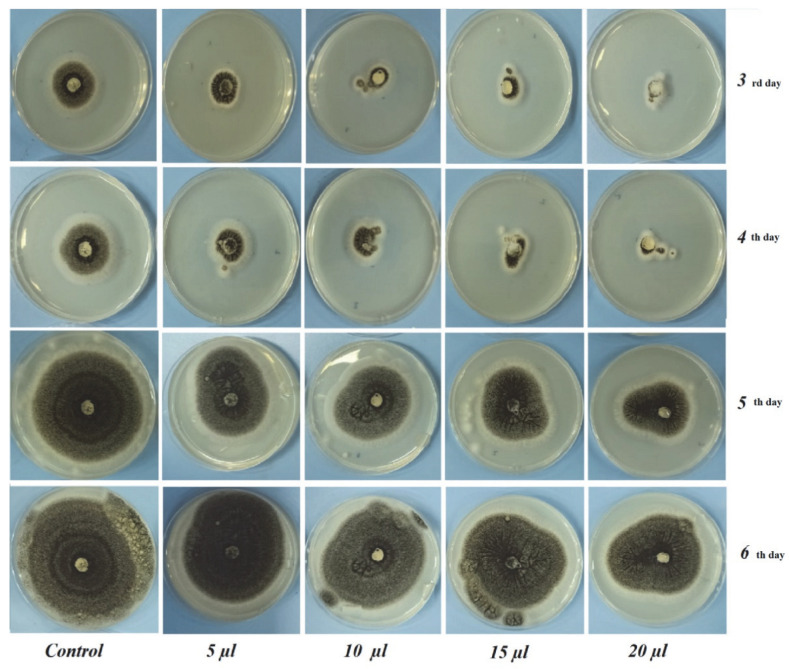
The antifungal effects of *C. bergamia* EO at different doses against *A. niger*.

**Figure 3:**
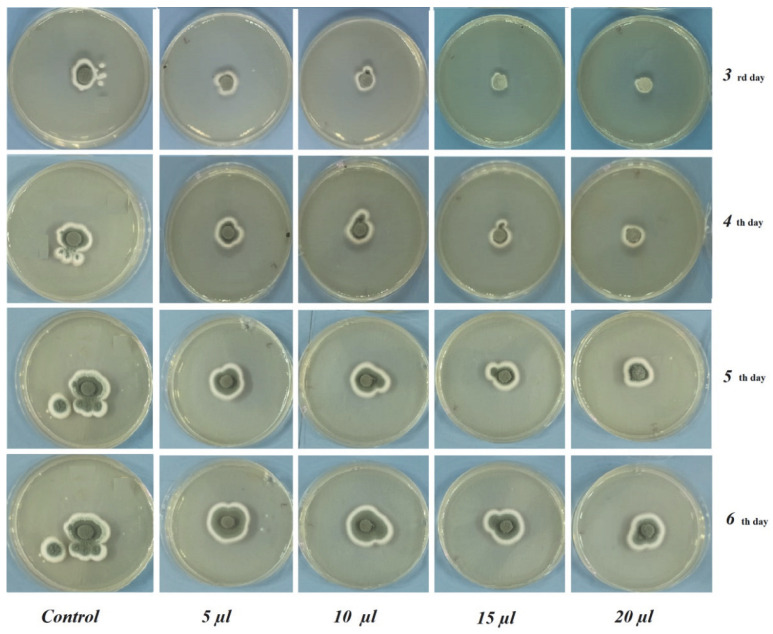
The antifungal effects of *C. bergamia* EO at different doses against *P. expansum*.

In the 6 days of incubation of *A. niger*, inhibition rates of 78.00 ± 0.00 mm at a 5 µL dose, 77.00 ± 2.82 mm at a 10 µL dose, 75.00 ± 0.00 mm at a 15 µL dose and 67.25 ± 0.35 mm at a 20 µL dose and 90.00 ± 0.00 mm growth of control were observed. In the 6 incubation days of *P. expansum*, inhibition rates of 32.00 ± 2.82 mm at 5 µL dose, 31.50 ± 0.00 mm at 10 µL dose, 27.00 ± 0.00 mm at 15 µL dose and 26.16 ±0.76 mm at 20 µL dose and 45.50 ± 2.12 mm of control fungi were observed. A higher antifungal effect on the part of *C. bergamia* EO was obtained against *P. expansum*. The experimental results suggested that increasing the doses (5, 10, 15, 20 μL/petri) of *C. bergamia* EO resulted in weaker growth of both *A. niger* and *P. expansum* (Figure 2 and Figure 3). 

**Figure 4:**
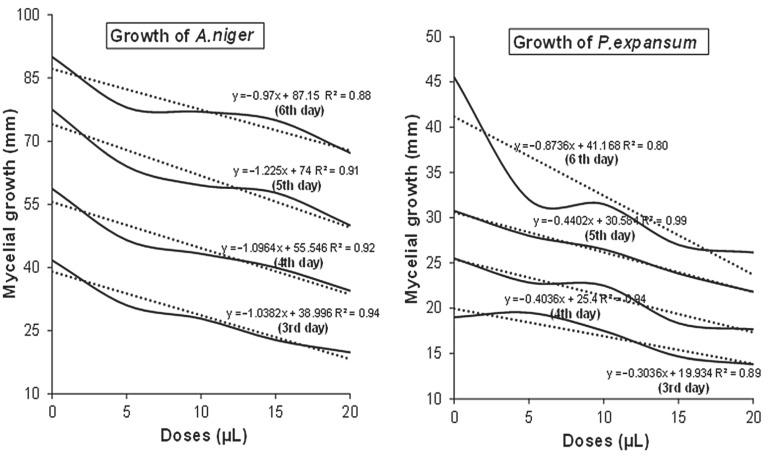
Plots of the dose (µL) of *C. bergamia* EO versus in vitro mycelial growth (mm) of *A. niger* and *P. expansum*, respectively (obtained by linear regression), showing a negative linear polynomial relationship between mycelial growth and doses, along with linear equations and R^2^ values.

In Figure 4, the results of the linear regression analysis are displayed as plots of the dose of *C. bergamia* EO versus in vitro mycelial growth of *A. niger* and *P. expansum*. It was determined that the relationship between dose application and fungal growth was linear and negative, based on the linear regression analysis, which was well explained by the high coefficients of determination (R^2^), which ranged from 0.80 and 0.99. The values of the linear determination coefficients obtained as a response to linear regression between fungal growth and dose application, suggested that the essential oil of *C. bergamia* possessed strong antifungal properties, which remarkably inhibited the growth of the fungal species considered.

### 3.3. FTIR Characterization of C. bergamia EO

**Figure 5:**
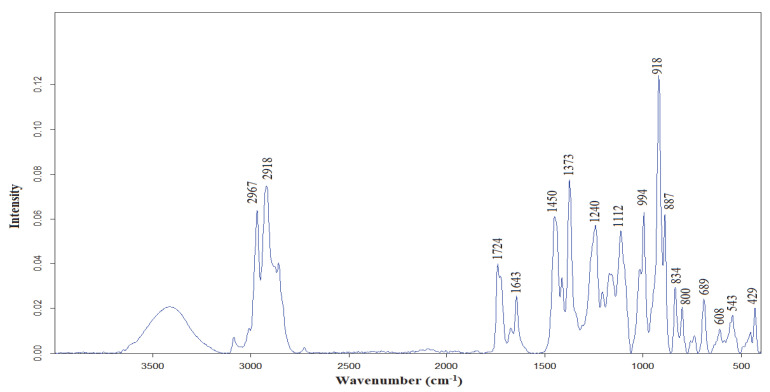
ATR-FTIR spectrum of *C. bergamia* EO essential oil at the spectral range of 4000–400 cm^−1^.

The ATR-FTIR spectrum of *C. bergamia* EO essential oil at the spectral range of 4000–400 cm^−1^ is presented in Figure 5. The major bands were observed at 2967, 2918, 1724, 1643, 1450, 1373, 1240, 1112, 994, 918, 887, 834 and 800 cm^−1^. In the previous studies, the FTIR band assignments, of a citrus species lemon essential oil, were presented [2]. Common FTIR bands were observed in the FTIR spectrum of *C. bergamia* EO. The band, with a peak point at 2967 cm^−1^, corresponded to –CH_3_ asymmetric and symmetric stretching vibrations [2]. The bands at 2918, 1724, 1643 cm^−1^ were due to the C–H stretching vibrations, the C=O stretching vibrations of ester groups, and ν(–C=C–, cis-) and δ(–OH) vibrations, respectively [16]. The band at 1450 cm^−1^ might be related to the bending vibrations of CH_2_ and CH_3_ of aliphatic groups [17]. The significant peaks at 1376 cm^−1^ and 887 cm^−1^ arose from C-H bending vibrations and (–HC = CH–, trans-) bending vibrations, respectively [16,18]. The significant bands at 994 and 918 cm^−1^ were probably due to δ(–HC=CH–, trans-) bending vibrations [16]. Lastly, the bands at 834 and 800 cm^−1^ corresponded to C–H stretching vibrations and C=C bending vibrations, respectively [2,19].

### 3.4. GC–MS Characterization of C. bergamia EO

The volatile compounds of *C. bergamia* essential oil obtained by hydro-distillation is presented in Table 4. These compounds were assigned by a comparison of GC–MS data with the commercial libraries (NIST27 and WILEY7) of the equipment. The volatile compounds presented in Table 4 explained 99.85% of all volatile ingredients in the composition of *C. bergamia* essential oil. The major volatile compounds were determined to be limonene (17.06%), linalool (46.34%) and linalyl acetate (17.69%). 

## 4. Discussion

The antimicrobial effects of essential oils suggest that their activity stems from their lipophilic properties. Interactions between antimicrobial compounds and cell membranes affect both lipid assembly and bilayer stability. Their mode of action occurs in the phospholipid bilayer, which is related to disruption of the cell membrane. This is caused by biochemical mechanisms catalyzed by the cell’s phospholipid bilayer. These processes include the inhibition of electron transport, protein translocation, phosphorylation steps, and other enzyme-dependent reactions [21].

Marotta et al. [15] evaluated the in vitro antimicrobial activity of various *C. bergamia* EOs against several strains of *L. monocytogenes*. Fisher and Phillips [22] demonstrated the antimicrobial activity of *C. bergamia* EO against *C. jejuni, E. coli* 0157, *L. monocyto-genes, B. cereus*, and *S. aureus*, in both direct oil and vapor forms. They stated that gram-positive bacteria were more susceptible in vitro than gram-negative bacteria. This may be due to the relatively impermeable outer membrane that surrounds gram-negative bacteria and restricts the diffusion of hydrophobic compounds [23,24]. 

Mycelium growth inhibition data, recorded at 6 days after inoculation at 25 ± 2 °C treated with *C. bergamia* EO at a 20 µL/petri concentration, showed the strongest mycelium growth inhibition of *P. expansum*. At 20 µL/petri concentrations, the most significant micelle inhibitory effect was observed for both fungi.

*C. bergamia* oil at a 0.8% *v/v* concentration inhibited the mycelium growth of pathogenic fungi. However, a powerful control could not be observed for *P. grisea* and *R. solani* [21].

Kulkarni et al. [9] investigated the antifungal activity of wild *C. bergamia* essential oil against the postharvest fungal pathogens, *Colletotrichum musae* and *Lasiodiplodia theobromae*, of banana fruit at concentrations of 1 mL to 10 mL. Their results exhibited 100% growth inhibition for both fungal pathogens at 4 mL.

Table 4 displays the chemical names, contents (%), and retention times (RTa) for the Rtx-5MS GC column, and the reported retention indices noted in the literature [20]. Monoterpene hydrocarbons, oxygenated monoterpenes and sequiterpene hydrocarbons constituted the volatile composition at percentages of 27.35%, 71.52 and 0.9%, respectively. This also accorded with some earlier observations, which showed that hydro-distillated *C. bergamia* essential oil involves limonene (32.29%), linalool (33.64%) and linalyl acetate (9.22%) [25]. However significant differences were observed between the limonene contents of *C. bergamia*. In accordance with the present results, previous studies showed that the major volatile compounds of *C. bergamia* essential oil were limonene, linalool and linalyl acetate [26].

The literature highlighted the importance of terpenes and terpenoids with respect to other EO components. Phenol ring structure-rich molecules were found to have considerable antibacterial activity, but enrichment with OH groups might increase their antibacterial properties [3]. The studies presented thus far provide evidence that the antifungal activities of essential oils are clearly related to their chemistry, including their chemical compounds, the percentage structure of each component, as well as their structure [9,27]. Kulkarni et al. [9] indicated that the chemical composition of wild bergamot (*Monarda fistulosa*) essential oil was determined by the GC–MS technique, and revealed that the main compounds of the essential oil, thymol, carvacrol and cinnamyl carbanilate, showed the best antifungal activities. Additionally, the antibacterial effect of terpenes is well known. They destroy the multi-layered structure of polysaccharides, fatty acids and phospholipids, penetrate the cell wall, and make the cytoplasmic membrane permeable. In bacteria, these events are associated with ion loss and decreased membrane potential, leading to breakdown of the proton pump and depletion of the ATP pool and lysis [15,28].

## 5. Conclusions

Volatile compound composition was determined using the GC–MS technique. The most abundant volatile constituents were determined to be limonene (17.06%), linalool (46.34%) and linalyl acetate (17.69%). The molecular fingerprint of the *C. bergamia* EO obtained by hydro-distillation was determined on the basis of FTIR spectroscopy. The essential oils from the *C. bergamia* peel provided anti-mycelial and anti-bacterial growth, and disrupted spore germination activity in the case of pathogenic fungi [29]. According to the results obtained, *C. bergamia* EO provides a good basis for the formulation of products, with potential effectiveness in terms of combatting bacteria and fungi. In addition, using smaller amounts of oil can have significant economic implications. These findings increase the opportunities and possibilities of exploiting essential oils as promising candidates for use in crop production systems, as alternative safe natural antifungal agents. In other words, the results suggested that bergamot peel essential oil could be used as a natural alternative for food preservation The findings from the current research may shed light with regard to the further use of *C. bergamia* peel EO in innovative food engineering applications as a means of maintaining food quality, ensuring food safety and improving food sustainability.

## Figures and Tables

**Table 1:** Zone of inhibition (mm) of *C. bergamia* EO against bacterial strains.

Species	0.5 µg/mL	1 µg/mL	2.5 µg/mL	5 µg/mL
*S. typhimurium*	9.25 ± 0.35 ^a^	10.87 ± 1.23 ^b^	11.50 ± 0.70 ^c^	11.50 ± 0.35 ^c^
*B. cereus*	8.50 ± 0.70 ^a^	12.00 ± 0.00 ^b^	12.50 ± 0.70 ^c^	12.50 ± 0.35 ^c^
*S. aureus*	10.00 ± 0.00 ^a^	10.50 ± 0.70 ^b^	10.50 ± 2.12 ^b^	11.50 ± 0.00 ^c^
*E. coli*	10.00 ± 0.00 ^a^	11.50 ± 0.70 ^b^	12.50 ± 0.70 ^c^	12.50 ± 0.00 ^c^
*L. monocytogenes*	10.00 ± 0.00 ^a^	12.75 ± 0.35 ^b^	13.00 ± 0.70 ^b^	13.50 ± 0.70 ^c^

All data are presented as mean ± standard deviation. ^a–c^: in each row, lowercase superscript letters indicate differences between doses of *C. bergamia* EO against each bacteria species. *p* < 0.05 was considered statistically significant.

**Table 2:** Inhibitory effect of *C. bergamia* EO against in vitro mycelial growth (mm) of *A. niger* at different incubation periods (*n* = 3).

Incubation Days	Control	5 µL	10 µL	15 µL	20 µL
3	41.66 ± 1.25 ^a^	31.00 ± 3.04 ^b^	27.83 ± 1.60 ^c^	22.75 ± 0.35 ^d^	19.83 ± 1.04 ^e^
4	58.66 ± 1.52 ^a^	46.5 ± 2.12 ^b^	43.25 ± 1.76 ^c^	40.00 ± 0.00 ^d^	34.50 ± 0.70 ^e^
5	77.5 ± 3.53 ^a^	64.00 ± 2.82 ^b^	59.5 ± 2.12 ^c^	57.75 ± 0.35 ^c^	50.00 ± 1.41 ^d^
6	90.00 ± 0.00 ^a^	78.00 ± 0.00 ^b^	77.00 ± 2.82 ^b^	75.00 ± 0.00 ^c^	67.25 ± 0.35 ^d^

All data are presented as mean ± standard deviation. ^a–e^: in each row, lowercase superscript letters indicate differences between doses at each incubation period. *p* < 0.05 was considered statistically significant.

**Table 3:** Inhibitory effect of *C. bergamia* EO against in vitro mycelial growth (mm) of *P. expansum* at different incubation periods (*n* = 3).

Incubation Days	Control	5 µL	10 µL	15 µL	20 µL
3	19.00 ± 0.00 ^a^	19.50 ± 1.50 ^b^	17.50 ± 0.00 ^c^	14.66 ± 0.76 ^d^	13.83 ± 0.76 ^e^
4	25.50 ± 0.35 ^a^	23.83 ± 2.56 ^b^	22.50 ± 0.00 ^c^	18.33 ± 0.76	17.66 ± 0.57 ^e^
5	30.75 ± 2.47 ^a^	28.00 ± 2.00 ^b^	26.50 ± 0.00 ^c^	23.83 ± 1.60 ^c^	21.83 ± 0.76 ^e^
6	45.50 ± 2.12 ^a^	32.00 ± 2.82 ^b^	31.50 ± 0.00 ^b^	27.00 ± 0.00 ^c^	26.16 ± 0.76 ^d^

All data are presented as mean ± standard deviation. ^a–e^: in each row, lowercase superscript letters indicate differences between doses at each incubation period. *p* < 0.05 was considered statistically significant.

**Table 4:** Composition (%), retention time (R.T.) and volatile compounds in the *C. bergamia* peel EO.

Content (%)	Chemical Name	RT ^a^	RI ^b^
0.14 ± 0.02	α-Thujene	8.541	924
0.57 ± 0.02	α-Pinene	8.701	932
0.45 ± 0.05	Sabinene	9.615	969
2.51 ± 0.03	β-Pinene	9.698	974
1.18 ± 0.07	Myrcene	9.983	988
0.04 ± 0.01	Octanal	10.234	998
0.16 ± 0.09	α-Terpinene	10.567	1014
17.06 ± 0.06	Limonene	10.947	1024
0.2 ± 0.04	Z-β-Ocimene	11.021	1032
0.54 ± 0.05	E-β-Ocimene	11.277	1044
4.11 ± 0.06	γ-Terpinene	11.604	1054
0.39 ± 0.06	Terpinolene	12.389	1086
46.34 ± 0.02	Linalool	13.046	1095
0.28 ± 0.03	Trans-sabinene hydrate	15.103	1098
2.41 ± 0.02	Exo-fenchol	15.522	1118
0.05 ± 0.09	Decanal	15.819	1201
0.08 ± 0.05	Acetic acid	15.969	1211
1.23 ± 0.01	Nerol	16.569	1227
0.56 ± 0.03	Z-citral	16.903	1235
17.69 ± 0.06	Linalyl acetate	17.414	1254
0.80 ± 0.05	Dimethoxy-(E)-citral	17.757	1338
0.95 ± 0.05	Neryl acetate	20.306	1359
1.13 ± 0.04	Geranyl acetate	20.822	1379
0.30 ± 0.05	Z-Caryophyllene	21.954	1408
0.27 ± 0.06	Cis-α-Bergamotene	22.290	1411
0.05 ± 0.07	Z-β -Farnesene	22.755	1440
0.36 ± 0.08	β-Bisabolene	24.126	1505
Total: 99.85	Total		
27.35	Monoterpene hydrocarbons		
71.52	Oxygenated monoterpenes		
0.98	Sesquiterpene hydrocarbons		
0	Oxygenated sesquiterpenes		

RT ^a^ Retention time of the compounds. RI ^b^ Reported retention indices in the literature [20].

## Data Availability

No new data were created or analyzed in this study. Data sharing is not applicable to this article.
